# Chloroform in Pneumonia

**Published:** 1856-03

**Authors:** 


*Chloroform in Pneumonia*—A Hungarian physician, Dr. Stohandl, reports three cases of pneumonia in which much benefit was derived from the inhalation of small quantities of chloroform (30 to 40 drops,) repeated several times a day. After each inhalation the symptoms were relieved; after four or six hours they again became aggravated, but were again relieved by a repetition of the inhalation. In from five to eight days a cure was effected.—*Revue de Therap. Med. Chirurg.*, Oct. 1, 1855, from *Ungar Zeitschrift.*

